# Postoperative XELOX therapy for patients with curatively resected high-risk stage II and stage III rectal cancer without preoperative chemoradiation: a prospective, multicenter, open-label, single-arm phase II study

**DOI:** 10.1186/s12885-019-6122-2

**Published:** 2019-09-18

**Authors:** Tsunekazu Mizushima, Masataka Ikeda, Takeshi Kato, Atsuyo Ikeda, Junichi Nishimura, Taishi Hata, Chu Matsuda, Taroh Satoh, Masaki Mori, Yuichiro Doki

**Affiliations:** 1 Multi-Center Clinical Study Group of Osaka University, Colorectal Group (MCSGO), 2-2 E2 Yamadaoka, Suita, Osaka, Japan; 2 Department of Gastroenterological Surgery, Osaka University Graduate School of Medicine, 2-2 E2 Yamadaoka, Suita, Osaka, 565-0871 Japan; 3 Division of Lower GI Surgery, Department of Surgery, Hyogo College of Medicine, 1-1 Mukogawa-cho, Nishinomiya, Hyogo Japan; 4 Department of Surgery, National Hospital Organization, National Hospital, 2-1-14 Hoenzakka, Chuo-ku, Osaka, Osaka Japan; 5 Department of Gastroenterological Surgery, Osaka Prefectural Hospital Organization, Osaka International Cancer Institute, 3-1-69 Otemae, Chuo-ku, Osaka, Japan; 6 Department of Surgery, Kansai Rosai Hospital, 3-1-69 Inabaso, Amagasaki, Hyogo Japan; 7 Department of Frontier-Science for Cancer and Chemotherapy, Osaka University Graduate School of Medicine, Yamadaoka 2-2, Suita, Osaka, Japan; 8 Department of Surgery and Science, Graduate School of Medical Science, Kyushu University, 3-1-1 Maidashi, Higashi-ku, Fukuoka, Japan

**Keywords:** Rectal cancer, Oxaliplatin, Capecitabine, XELOX, Adjuvant chemotherapy

## Abstract

**Background:**

Preoperative 5-FU-based chemoradiation is currently a standard treatment for advanced rectal cancer, particularly in Western countries. Although it reduced the local recurrence, it could not necessarily improve overall survival. Furthermore, it can also produce adverse effects and long-term sphincter function deficiency. Adjuvant oxaliplatin plus capecitabine (XELOX) is a recommended regimen for patients with curatively resected colon cancer. However, the efficacy of postoperative adjuvant therapy for rectal cancer patients who have not undergone preoperative chemoradiation remains unknown. We aimed to evaluate the efficacy of surgery and postoperative XELOX without preoperative chemoradiation for treating rectal cancer.

**Methods:**

We performed a prospective, multicenter, open-label, single arm phase II study. Patients with curatively resected high-risk stage II and stage III rectal cancer who had not undergone preoperative therapy were treated with a 120 min intravenous infusion of oxaliplatin (130 mg/m^2^) on day 1 and capecitabine (2000 mg/m^2^/day) in 2 divided doses for 14 days of a 3-week cycle, for a total of 8 cycles (24 weeks). The primary endpoint was 3-year disease-free survival (DFS).

**Results:**

Between August 2012 and June 2015, 60 men and 47 women with a median age was 63 years (range: 29–77 years) were enrolled. Ninety-three patients had Eastern Cooperative Oncology Group performance status scores of ‘0’ and 14 had scores of ‘1’. Tumors were located in the upper and lower rectums in 54 and 48 patients, respectively; 8 patients had stage II disease and 99 had stage III. The 3-year DFS was 70.1% (95% confidence interval, 60.8–78.0%) and 33 patients (31%) experienced recurrence, most commonly in the lung (16 patients) followed by local recurrence (9) and hepatic recurrence (7).

**Conclusions:**

Postoperative XELOX without preoperative chemoradiation is effective for rectal cancer and provides adequate 3-year DFS prospects.

**Trial registration:**

This clinical trial was registered in the University Hospital Medical Information Network registry system as UMIN000008634 at Aug 06, 2012.

## Background

Chemotherapy and radiotherapy are both used as adjuvant therapies before and after the curative resection of advanced rectal cancer, and aim to prevent recurrence and extend survival by eliminating micrometastases. Postoperative adjuvant chemotherapy for colon cancer has been shown to reduce recurrence and extend survival in large-scale clinical trials. Studies of the efficacy of 5-fluorouracil (5-FU)-based postoperative adjuvant chemotherapy have been performed since the 1980s; based on the results of clinical trials such as the MOSAIC, NSASBPC-04, and NO16968 trials, the current standard treatment involves either combination 5-FU/oxaliplatin-based therapies (5-FU, leucovorin, and oxaliplatin [FOLFOX] or [FLOX]) or alternatively oral fluoropyrimidine capecitabine and oxaliplatin (XELOX) [1–3].

However, evidence for the efficacy of postoperative-only adjuvant chemotherapy in patients with rectal cancer is sparse. The only published data concerning postoperative adjuvant chemotherapy in patients who did not receive preoperative treatments such as chemoradiation are those concerning tegafur/uracil with or without radiotherapy; no such studies involving oxaliplatin have been performed [4–6]. In a study of postoperative adjuvant chemotherapy after preoperative chemoradiation, Hong et al. found that disease-free survival (DFS) for patients treated with postoperative FOLFOX after preoperative 5-FU-based chemoradiotherapy and total mesorectal excision (TME) was 71.6%, which was significantly better than the 62.9% 3-year DFS rate for patients treated with 5-FU/*l*-leucovorin [7]. However, adjuvant radiotherapy has not been widely used for a long time in Japan, and TME with lateral lymph node dissection or TME with 5-FU based adjuvant chemotherapy has been the standard treatment [8]. The efficacy of postoperative-only adjuvant therapy for patients with rectal cancer who did not receive preoperative chemoradiation remains unknown [9, 10]. Therefore, we conducted a clinical trial to evaluate the efficacy of surgery and postoperative adjuvant XELOX therapy without preoperative chemoradiation for treating rectal cancer.

## Methods

### Eligibility criteria

Eligible patients were those aged 20 years or older with histologically proven rectal cancer; stage II with at least one risk factor of recurrence (such as T4, vessel or lymphatic invasion, perineural invasion, perforation, obstruction, lower histological grade, or higher preoperative carcinoembryonic antigen levels) or stage III [11]; curative resection (R0) with lymph node dissection; Eastern Clinical Oncology Group performance status score of 0 or 1; no prior chemotherapy or radiotherapy; adequate food intake orally; adequate function of vital organs (white blood cell count ≥3000/mm^3^, neutrophil count ≥1500/mm^3^, platelet count ≥100,000/mm^3^, hemoglobin ≥9.0 g/dL, serum aspartate aminotransferase and alanine aminotransferase ≤2.5-fold the institutional upper limit of normal (ULN), serum total bilirubin ≤2.5-fold the ULN, and serum creatinine ≤1.5-fold the ULN). The rectum was defined as the area between the promontorium and the upper edge of the anal canal according to the Japanese Classification of Colorectal Carcinoma, 8th edition (second English edition); moreover, the Union for International Cancer Control TNM Classification of Malignant Tumors (7th edition) was used for staging. Postoperative adjuvant chemotherapy as started within 8 weeks post-surgery. Written informed consent was obtained from all patients before enrollment. Patients were excluded if they had unresolved postoperative complications, synchronous or metachronous (within 5 years) malignancies other than carcinoma in situ, severe paresthesia or dysesthesia with dysfunction, a past history of severe drug allergy, active infection, severe mental disorder, uncontrollable diabetes or hypertension, interstitial pneumonia or pulmonary fibrosis, intestinal palsy or obstruction, or severe heart disease. Moreover, women who were pregnant or lactating, patients who were sexually active and unwilling to use contraception, and subjects in poorer physical conditions (as determined by the primary physician) were also excluded.

### Study design and treatment

This open-label, single-arm phase II study involving 19 institutions aimed to evaluate the safety and efficacy of adjuvant XELOX therapy for patients with curatively resected high-risk stage II and stage III rectal cancer. The study protocol was approved by Osaka University Clinical Research Review Committee. This clinical trial was registered in the University Hospital Medical Information Network registry system as UMIN000008634 at Aug 06, 2012.

Enrolled patients commenced the XELOX protocol treatment within 8 weeks post-surgery. The protocol treatment consisted of a 120 min intravenous infusion of oxaliplatin 130 mg/m^2^ on day 1 and oral capecitabine 2000 mg/m^2^/day per day in 2 divided doses for 14 days of a 3-week cycle, for a total of 8 cycles (24 weeks) or until unacceptable toxicity occurred.

Treatment was postponed for a maximum of 42 days if any of the following criteria were not met within 24 h of the start of each course: neutrophil count ≥1500/mm^3^, platelet count ≥75,000/mm^3^, persistent grade ≤ 1 peripheral sensory neuropathy, grade ≤ 1 hand-foot syndrome, and any other parameter as decided by the attending physician. Dose modifications were based on the most severe adverse events observed during the previous treatment cycle, and were performed according to previously reported criteria [12].

After the completion of the protocol treatment, surveillance for recurrence was performed via outpatient medical interviews and measurement of the tumor markers carcinoembryonic antigen and CA19–9 every 3 months. Diagnostic imaging using radiography or computed tomography of the chest, or via ultrasonography or computed tomography of the abdomen, was also performed every 6 months. If recurrence was suspected, the most appropriate diagnostic modality was used to confirm its occurrence.

### Primary and secondary endpoints

The primary endpoint was the 3-year DFS. Secondary endpoints were the safety profile (rate and severity of adverse events), treatment completion rate, and relative dose intensity.

### Statistical analysis

The reported 3-year DFS rates were between 62 and 69% in patients who underwent 5-FU based preoperative chemoradiotherapy and TME [13–15], which are the standard procedures for treating advanced rectal cancer. Ninety-five patients were required to test the null hypothesis versus the alternative hypothesis with a 1-sided α level of 0.05 and β level of 0.1 when the critical value of 3-year DFS was 60% and the expected value was 65%. The total number of patients required for this study was thus calculated to be 95. The JMP® 10 software (SAS Institute Inc., Cary, NC, USA) was used for all statistical analyses.

## Results

### Patient characteristics

From 19 of the 22 institutions participating in this study, 107 patients who met the inclusion criteria were enrolled between October 2010 and June 2014. The patients’ characteristics are shown in Table 1. The median patient age was 63 years (range: 29–77 years), among whom there were 60 men and 47 women. The performance status score was ‘0’ in 93 patients and ‘1’ in 14. Low anterior resection was performed in most patients (74%), although other procedures including abdominoperineal resection, high anterior resection, intersphincteric resection, and Hartmann’s procedure were also used. Despite its recommendation by the Japanese guidelines, lateral lymph node dissection was performed in only 16 of the 48 patients with lower rectal cancers (33.3%).

**Table 1:** Baseline characteristics of patients

	*n* = 107
Age, years (range)	63 (29–77)
Sex	
Male	60 (56%)
Female	47 (44%)
Eastern Clinical Oncology Group (ECOG) paformance status	
0	93 (87%)
1	14 (13%)
Main location of the tumor	
Rectosigmoid	5 (5%)
Upper rectum	54 (50%)
Lower rectum	48 (45%)
Surgical approach	
Laparoscopic	77 (72%)
Open	30 (28%)
Surgical procedure	
HAR	10 (9%)
LAR	79 (74%)
ISR	4 (4%)
Hartmann’s procedure	2 (2%)
APR	12 (11%)
Lateral lymph node dissection by the main location of the tumor	19 (18%)
Upper rectum	3
Lower rectum	16
Operation time, minutes (range)	311 (122–914)
Intraopelative bleeding, ml (range)	45 (0–2100)
Stoma creation	47 (44%)
Postoperative complications	18 (17%)
Histology	
pap/tub1/tub2	1/33/71
por1/por2/muc/sig	1/1/0/0
Pathological T category	
T1	7 (7%)
T2	19 (18%)
T3	66 (62%)
T4a	12 (11%)
T4b	3 (3%)
Pathological N category	
N0	8 (8%)
N1a	24 (22%)
N1b	29 (27%)
N2a	21 (20%)
N2b	25 (23%)
Pathological stage	
IIA	5 (5%)
IIB	3 (3%)
IIIA	17 (16%)
IIIB	57 (53%)
IIIC	25 (23%)

*HAR* high anterior resection, *LAR* low anterior resection, *ISR* intersphincteric resection, *APR* abdominoperineal resection

The median operation time was 311 min and the median intraoperative bleeding volume was 45 mL; the vast majority of patients (84%) underwent a rectal washout prior to transection, which is important to prevent local recurrence caused by residual cancer cells. A stoma was inserted for 47 patients (44%), including 33 diverting ileostomies, and postoperative complications occurred in 18 patients (17%).

The histopathological diagnosis was papillary adenocarcinoma or well-to-moderately differentiated tubular adenocarcinoma in almost all patients (105); 8 patients had stage II disease and 99 had stage III (Table 1).

### Dose intensity and treatment compliance

The patients were treated with a median of 8 courses (range, 1–8 courses). The median doses of capecitabine and oxaliplatin were 180,000 mg/m^2^ and 788.1 mg/m^2^, respectively, and their respective relative dose intensities were 83.7 and 82.4%, which are levels similar to those previously reported for postoperative adjuvant chemotherapy for colon cancer (Table 2).

**Table 2:** Dose intensity and treatment compliance

	*n* = 107
Total dose, median (range), mg/m^2^	
Capecitabine	180,000 (1008–292,174)
Oxaliplatin	788.1 (26.6–1079.6)
Relative dose intensity, median (range), %	
Capecitabine	83.7 (3.5–130.4)
Oxaliplatin	82.4 (3.8–198.8)
Course of treatment, median (range)	8 (1–8)

### Efficacy

**Fig. 1:**
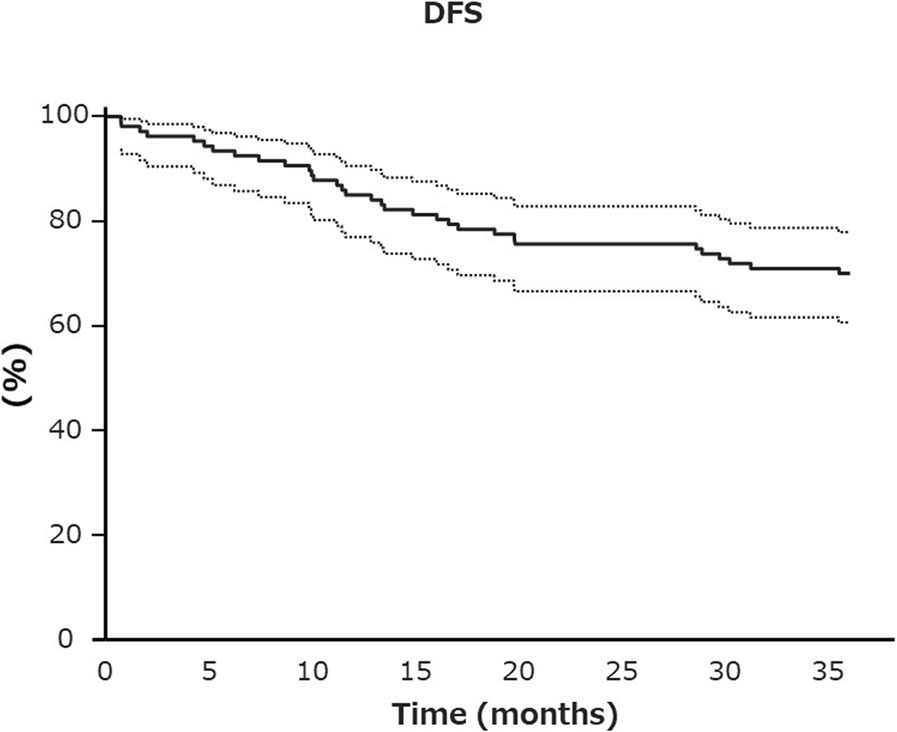
Kaplan-Meier curves showing disease-free survival (DFS). The 3-year DFS rate was 70.1% (60.8–78.0%)

The median follow-up was 49.3 months (4.7–73.6 months), while the 3-year DFS was 70.1% (60.8–78.0%); these results were favorable and exceeded the anticipated value of 65% (Fig. 1). Thirty-three patients (31%) experienced recurrence; the most common site of initial recurrence was the lungs (15%), followed by local recurrence in 8% and the liver in 7%. Local recurrence was intrapelvic in 4 patients, at the anastomosis site in 3, and in pelvic lymph nodes in 2 (Table 3).

**Table 3:** Patterns of recurrence

	n (%)
Lung	16 (15%)
Local recurrence	9 (8%)
Anastomosis	3
Pelvis	4
Pelvic lymph nodes	2
Liver	7 (7%)
Peritoneal dissemination	1 (1%)
Paraaortic lymph nodes	1 (1%)
Sigmoid colon	1 (1%)
Ovary	1 (1%)
Total^a^	33 (31%)

^a^Two patients developed recurrence in lung and liver, and one patients developed recurrence in lung and local recurrence

### Safety

Common hematotoxic adverse events included anemia (48%), leukopenia (42%), neutropenia (39%), and thrombocytopenia (43%). Peripheral sensory neuropathy (64%), nausea (48%), liver dysfunction (increased aspartate aminotransferase and alanine aminotransferase in 46 and 35%, respectively), and hand-foot syndrome (30%) were comparatively frequent non-hematotoxic adverse events. However, the rates of grade ≥ 3 hematotoxic and non-hematotoxic events were both < 10% (Table 4).

**Table 4:** Adverse events

n (%)	Any grade	Grade 3	Grade 4
Anemia	51 (48%)	0	1 (1%)
Neutropenia	42 (39%)	8 (7%)	1 (1%)
Leukopenia	45 (42%)	0	1 (1%)
Thrombocytopenia	46 (43%)	2 (2%)	1 (1%)
AST increased	49 (46%)	0	0
ALT increased	37 (35%)	1 (1%)	0
Hand-foot syndrome	32 (30%)	2 (2%)	0
Peripheral sensory neuropathy	69 (64%)	10 (9%)	0
Loss of appetite	28 (26%)	3 (3%)	0
General fatigue	25 (23%)	2 (2%)	0
Nausea	26 (24%)	2 (2%)	0
Vomiting	8 (7%)	3 (3%)	0
Diarrhea	19 (18%)	4 (4%)	0
Allergy	6 (3%)	2 (2%)	0

## Discussion

In this study, the 3-year DFS after surgery and postoperative adjuvant XELOX therapy in patients who received no preoperative chemoradiation was 70.1%. This result was not inferior to that of the standard rectal cancer treatment of delivering chemoradiation before surgery and suggest the efficacy of adding oxaliplatin to 5-FU based adjuvant chemotherapy. Lung metastasis (15%, the most common type of recurrence) and liver metastases (7%) accounted for almost all cases of distant recurrence, while local recurrence was observed in 8% of the patients.

Local recurrence after rectal cancer surgery was formerly common given the anatomy of the colorectal region. However, the incidences of recurrence have decreased since the introduction of TME in the late 1980s; hence, the lung and liver are now the most common sites of recurrence [16, 17]. Preoperative 5-FU-based chemoradiation is currently a standard treatment as a means of further controlling local recurrence, particularly in Western countries. A Dutch group compared surgery alone to that plus preoperative radiation and found that the 2-year cumulative local recurrence rates were 8.2 and 2.4%, respectively, demonstrating that the local recurrence rate was significantly reduced when preoperative radiation was included [16]. However, the efficacy of preoperative radiation has not been demonstrated in terms of long-term outcomes; the 10-year distant metastasis rates are 28% for surgery alone and 25% for surgery plus preoperative radiation, with overall survival (OS) rates of 49 and 48%, respectively [18]. A German group also reported that preoperative chemoradiation is effective in suppressing local recurrence, with a 5-year cumulative local recurrence rate of 13% in patients receiving postoperative chemoradiation but of only 6% in those receiving preoperative chemoradiation. However, that study did not demonstrate efficacy in terms of DFS or OS, as the 10-year DFS was 67.8% with postoperative chemoradiation and 68.1% with preoperative chemoradiation, while the corresponding 10-year OS rates were 59.9 and 59.6%, respectively [14]. Although preoperative chemoradiation reduces the local recurrence rate, the data collectively indicates that this therapy alone does not contribute to improving OS.

In contrast to Western countries, the standard treatment for rectal cancer in Japan consists of surgery (rectal resection or amputation and lymph node dissection) without preoperative chemoradiation. Lateral lymph node dissection is also recommended for advanced lower rectal cancers. The reported rate of lateral lymph node metastasis for lower rectal cancers that have invaded beyond the muscularis propria but are negative for lymph node metastasis within the mesorectum is 18.4%, with this rate rising to 23.5% if lymph node metastasis within the mesorectum is present; lateral lymph node dissection is expected to reduce the risk of intrapelvic recurrence by 50.3% [19]. In the JCOG0212 trial, TME alone has failed to show non-inferiority to TME plus lateral lymph node dissection, even in patients with no evident lateral lymph node metastasis on preoperative diagnostic imaging. Moreover, TME with lateral lymph node dissection reduced local recurrence (pelvic lymph node recurrence) [20].

Chemoradiation in combination with drugs other than 5-FU and the addition of postoperative adjuvant chemotherapy to preoperative chemoradiation was investigated for its efficacy in extending survival and improving long-term prognosis, as was the addition of oxaliplatin for pre- or postoperative therapy. Hofheinz et al. showed the superior results of chemoradiotherapy with capecitabine. 5-year overall survival in the capecitabine group was non-inferior to that in the fluorouracil group and post-hoc test showed the superiority of the capecitabine group. 3-year DFS was 75% in the capecitabine group and 67% in the fluorouracil group [15]. The CAO/ARO/AIO-94 group compared patients who underwent surgery following preoperative chemoradiation with 50.4 Gy plus 5-FU and who subsequently received postoperative chemotherapy with 5-FU alone, with those who underwent surgery following preoperative chemoradiation with 50.4 Gy plus 5-FU/oxaliplatin and subsequently received postoperative chemotherapy with 5-FU/oxaliplatin. They found that the 3-year DFS was 71.2% in the 5-FU arm and 75.9% in the 5-FU/oxaliplatin arm, suggesting that oxaliplatin may provide an additional benefit [21].

Preoperative chemoradiation for rectal cancer has been reported to cause long-term anal sphincter dysfunction [22]. The rate of fecal incontinence at 5 years after preoperative chemoradiation and surgery has increased significantly from 38 to 62% because of the inclusion of radiotherapy; this tendency is particularly pronounced in patients with tumors located 5–10 cm from the anal verge. The rates of bloody stool (3% following surgery alone compared with 11% following preoperative chemoradiation plus surgery) and of mucous and bloody stool (15 and 27% of the corresponding patients, respectively) had also increased significantly because of the inclusion of radiotherapy, thereby affecting the patients’ daily activities [23]. The additional use of radiotherapy has also been reported to increase the risk of secondary cancer in organs within or adjacent to the irradiation field [24].

Attempts have also been made to treat rectal cancer patients with preoperative chemotherapy, including with oxaliplatin, with the aim of preventing recurrence (including postoperative distant metastasis); the goal was to introduce intensive preoperative chemotherapy while avoiding the adverse events associated with preoperative radiation [25, 26]. In a study by Uehara et al. [20], 84% of patients completed the treatment plan of 4 cycles of preoperative XELOX plus bevacizumab followed by TME or tumor-specific mesorectal excision; 13% achieved pathological complete response, 90% had R0 resection, 60% achieved T downstaging, and 83.3% achieved N downstaging. In another study by Hasegawa et al. [21], the protocol completion rate was 72%, the pathological complete response rate was 4.3%, the R0 resection rate was 100%, and T and N downstaging rates were 69.6 and 78.9%, respectively. Both these studies suggested that this method may be effective for suppressing distant metastasis via R0 surgery and preoperative downstaging. The combination of postoperative adjuvant XELOX therapy (the efficacy of which was demonstrated in this study), intensive preoperative chemotherapy to control distant metastasis, and lateral lymph node resection for local control thus has the potential to become a new standard treatment for rectal cancer that preserves anal sphincter function.

The limitation of this study was that it was single-arm trial that lacked a control arm such as a surgery-only arm. Because the prognosis of patients with advanced rectal cancer is worse than that of patients with colon cancer, such patients generally do not undergo surgery without adjuvant therapy. Future studies ought to compare patients undergoing preoperative chemoradiation to those undergoing intensive postoperative chemotherapy.

## Conclusion

We have demonstrated that postoperative XELOX therapy without preoperative chemoradiation is an effective treatment method for rectal cancer that offers the prospect of adequate 3-year DFS.

## Data Availability

The datasets generated and analysed during the current study are not publicly available due to consent from participants but are available from the corresponding author on reasonable request.

## Abbreviations

5-FU: 5-fluorouracil
DFS: Disease-free survival
OS: Overall survival
TME: Total mesorectal excision
ULN: Upper limit of normal
